# Validation of coding algorithms for identifying people with viral hepatitis using claims data according to different standard references

**DOI:** 10.1186/s12879-022-07212-w

**Published:** 2022-03-04

**Authors:** Ming-Jen Sheu, Tsung-Wei Chin, Fang-Ping Ku, Chung-Yi Li, Sheng-Tun Li, Tsung-Hsueh Lu

**Affiliations:** 1 Division of Gastroenterology and Hepatology, Chi Mei Medical Center, Tainan, Taiwan; 2 Department of Public Health, College of Medicine, National Cheng Kung University, No. 1, Dah Hsueh Road, East District, Tainan, 701 Taiwan; 3 Department of Healthcare Administration, College of Medical and Health Science, Asia University, Taichung, Taiwan; 4 Department of Public Health, College of Public Health, China Medical University, Taichung, Taiwan; 5 Department of Industrial and Information Management, College of Management, National Cheng Kung University, Tainan, Taiwan; 6 Center for Innovative FinTech Business Models, National Cheng Kung University, Tainan, Taiwan

**Keywords:** Hepatitis B virus, Hepatitis C virus, *International Classification of Disease (ICD)*, Claims data, Algorithms, Validation study

## Abstract

**Background:**

To assess the performance of various coding algorithms for identifying people with hepatitis B virus (HBV) and hepatitis C virus (HCV) using claims data according to different reference standards (RSs) and study periods (SPs).

**Methods:**

A proportional random sampling of 10,000 patients aged ≥ 20 years in a health care system in Southern Taiwan were enrolled as study participants. We used three hierarchical RSs (RS1: having positive results of laboratory tests; R2: having RS1 or having prescriptions of anti-HBV or anti-HCV medications; R3: having R1 or R2 or having textual diagnosis recorded in electrical medical records) with three SPs (4-, 8-, and 12-years) to calculate positive predictive value (PPV) and sensitivity (Sen) of 6 coding algorithms using HBV- and HCV-related *International Classification of Disease Tenth Revision Clinical Modification* (*ICD-10-CM*) codes in Taiwan National Health Insurance claims data for years 2016–2019.

**Results:**

Of 10,000 enrolled participants, the number of participants had confirmed HBV and HCV was 146 and 165, respectively according to RS1 with 4-years SP and increased to 729 and 525, respectively according to RS3 with 12-years SP. For both HBV and HCV, the PPV was lowest according to RS1 and highest according to RS3. The longer the SP, the higher the PPV. However, the Sen was highest according to RS2 with 4-years SP. For both HBV and HCV, the coding algorithm with highest PPV and Sen was “ ≥ 3 outpatient codes” and “ ≥ 2 outpatient or ≥ 1 inpatients codes,” respectively.

**Conclusions:**

In conclusion, using different RSs with different SPs would result in different estimation of PPV and Sen. To achieve the best yield of both PPV and Sen, the optimal coding algorithm is “ ≥ 2 outpatients or ≥ 1 inpatients codes” for identifying people with HBV or HCV.

**Supplementary Information:**

The online version contains supplementary material available at 10.1186/s12879-022-07212-w.

## Background

An increasing need of using real world data to assess the effectiveness and safety of medications was suggested [1–3]. With regard to viral hepatitis, several recent studies have used administrative data in healthcare system to examine the effectiveness and safety of anti-viral medications [4–8]. One critical step in using the administrative data is the use of the *International Classification of Diseases* (*ICD*) codes for identifying people with a given disease, either as the main health outcome or as a covariate [9, 10]. The recommendations on the reporting of studies checklist item 6.2 in *Reporting of studies conducted using observational routinely-collected health data* (*RECORD*) states that “any validation studies of the codes or algorithms used to select the population should be referenced.” [11].

One important reason of conducting validation studies for coding algorithms is that the *ICD* codes in reimbursement claims or bills for outpatient medical services are assigned by physicians, who might not be very familiar with appropriate *ICD* codes that are not in their specialties. In some occasions, the *ICD* codes are assigned by clerks who have not had contact with the patient and may be entering them to address other administrative hurdles. For example, to get an outpatient laboratory test accepted for payment requires that there is a tentative *ICD* diagnosis and tends to be less accurate than one made by a physician after medical evaluation.

However, only few studies have examined the validity of algorithms using *ICD* codes to identify people with hepatitis B virus (HBV) or hepatitis C virus (HCV) infection [12–16]. There was one national study examined the validity of using *ICD* codes recorded in Taiwan National Health Insurance (NHI) claims data for identifying people with HBV or HCV [16]. However, that study assessed only one coding algorithm and used only the results of laboratory tests as reference standard (RS) [16]. Nevertheless, the four US viral hepatitis coding validation studies included also the pharmacy data and textual diagnosis recorded in electronic medical record (EMR) as components of RS [12–15]. The reason of including the textual diagnosis as RS was that people with HBV or HCV had laboratory tests with positive results several years ago, so the physicians will not repeat the laboratory tests again in recent years. If we confined the RS to results of laboratory tests only, these people with HBV or HCV would not be confirmed by the RS using only results of laboratory tests.

Another methodological issue was that different studies implemented different study periods (SPs) for RSs, which was 7-years [12], 3-years, [13] 11-years [14], and 7-years [15], respectively in four US studies. Little is known whether the calculated validity indicators (positive predictive value [PPV] and sensitivity [Sen]) might be different if different SPs were executed. Therefore, this study aimed to assess the performance of various coding algorithms using *ICD* codes in Taiwan NHI claims data according to different RSs with different components and different SPs for identifying people with HBV and HCV.

## Methods

### Protocol

This study was reported in accordance with STARD 2015 guideline [17].

### Study design and setting

This study is a retrospective study, in which the index test (coding algorithms) and RS were collected retrospectively. The study was conducted at the Chi Mei health care system in Tainan, Taiwan. The Chi Mei health care system is the largest integrated health care system in southern Taiwan and includes one e medical center (Yongkang), one regional hospital (Liouying), and one district hospital (Jiali). This study was approved by the Institutional Review Board of the Chi Mei Medical Center (number: 10901-015).

### Participants

A proportional random sampling of 10,000 patients aged ≥ 20 years who had at least four visits (any causes) to the Chi Mei health care system in 2016 were enrolled as study participants. The *ICD Tenth Revision Clinical Modification* (*ICD-10-CM*) was implemented in Taiwan in January first, 2016. Of 10,000 patients sampled, 60% from Yongkang medical center, 25% from Liouying regional hospital, and 15% from Jiali district hospital according to the proportion of total patient visits in three hospitals. The sample size of 10,000 people was estimated according to the prevalence of HBV and HCV in Taiwan and for further sub-population analyses.

The first reason of confining to people who had at least four visits a year was to enroll people whose ‘usual-care’ hospital was in Chi Mei health system. Therefore, the enrolled people had sufficient information recorded in EMR for RSs. The second reason was that the main purpose of most studies using real world data is to assess the effectiveness and safety of medications for people with specific chronic disease. The target population of this study was those with chronic disease and need regular visits in a given hospital. The number of patients who had at least four visits in 2016 among all patients was 100,624/260,566 (39%) in Yongkang medical center, 50,749/118,550 (43%) in Liouying regional hospital, and 26,201/62,295 (42%) in Jiali district hospital, about two fifths of total patients.

### Reference standards

We used three hierarchical RSs to define people with HBV or HCV. RS1: having positive results of HBV surface antigen (HBsAg), e-antigen (HBeAg), or HCV antibody (Anti-HCV) tests; RS2: having RS1 or having prescriptions of anti-HBV or anti-HCV medications reimbursed by the Taiwan NHI; RS3: having RS1 or RS2 or textual diagnosis of HBV or HCV in the discharge summary for inpatient hospitalization or in the problem list and past history for outpatient visits. A query system (Hyperion) was used to review EMR for each participant 4-years (2016–2019), 8-years (2012–2019), and 12-years (2008–2019).

The reason of using different RSs with different SPs was that many patients have received the laboratory tests in other hospitals or several years ago in ‘usual-care’ hospital, therefore, the physicians would not repeat the tests again. The Taiwan NHI administration would not reimburse the duplicated tests and drug prescriptions if the patients had the same tests and prescriptions recently in other hospitals [18]. If we used only results of laboratory tests for short SP, many patients with HBV or HCV would be misclassified as without HBV or HCV. As the status of HBV or HCV is lifelong; therefore, the RS should be more comprehensive and the SP should be as long as possible to avoid the misclassification.

### Index test (coding algorithms)

The *ICD-10-CM* codes for HBV were *B16.0, B16.1, B16.2, B16.9, B17.0, B18.0, B18.1, B19.1, Z22.51* and the codes for HCV were *B17.1, B18.2, B19.2, Z22.52*. We developed the following 6 coding algorithms using *ICD-10-CM* codes in claims data for years 2016–2020:

1.  ≥ 1 outpatient codes
2.  ≥ 2 outpatient codes
3.  ≥ 3 outpatient codes
4.  ≥ 2 outpatient or ≥ 1 inpatient codes
5.  ≥ 3 outpatient or ≥ 1 inpatient codes
6.  ≥ 4 outpatient or ≥ 1 inpatient codes

### Analysis

We calculated PPV and Sen to assess the performance of 6 coding algorithms for identifying people with HBV or HCV infection. PPV is defined as the probability of confirmed HBV or HCV in patients with positive coding algorithm, ie, a/(a + b) in the following 2 by 2 table. The definition of Sen is defined as proportion of people with confirmed HBV or HCV who have positive coding algorithm, ie, a/(a + c) in the following 2 by 2 table. To reveal the stability of estimation of PPV and Sen we calculated 95% confidence intervals (95% CI) for both indicators.

**Table:** 

	Reference standard for HBV or HCV	
	Confirmed	Not confirmed
Coding algorithm		
Positive	a (True positive)	b (False positive)
Negative	c (False negative)	d (True negative)

## Results

The number of enrolled participants been confirmed to have HBV or HCV varied greatly depends on the RS and SP used. Of 10,000 enrolled participants, the number of participants had confirmed HBV and HCV was 146 and 165, respectively according to RS1 with 4-years SP and increased to 729 and 525, respectively according to RS3 with 12-years SP (Table 1).

**Table 1:** Study participants been confirmed as having hepatitis B virus (HBV) or hepatitis C virus (HCV) infection according to 3 reference standards (RSs) and 3 study periods

Study	All participants		RS1 confirmed		RS2 confirmed		RS3 confirmed	
Period (years)	No.	%	No.	%	No.	%	No.	%
*HBV*								
4 (2016–2019)	10,000	100.0	146	1.5	247	2.5	537	5.4
8 (2012–2019)	10,000	100.0	212	2.1	336	3.4	650	6.5
12 (2008–2019)	10,000	100.0	297	3.0	394	3.9	729	7.3
*HCV*								
4 (2016–2019)	10,000	100.0	165	1.7	224	2.2	407	4.1
8 (2012–2019)	10,000	100.0	336	3.4	375	3.8	498	5.0
12 (2008–2019)	10,000	100.0	498	5.0	393	3.9	525	5.3

RS1: having positive results of laboratory test HBsAg or HBeAg or Anti-HCV

RS2: having RS1 or having prescriptions of anti-HBV or anti-HCV medications

RS3: having RS1 or RS2 or having HBV or HCV textual diagnosis recorded in electrical medical records

Table 2 illustrates the PPV and the Sen for 6 coding algorithms for identifying people with HBV according to 3 RSs and 2 SPs and Additional file 1: Table S1 presents the numerators and denominators for each PPV and Sen. The PPV was lowest according to RS1 and highest according to RS3. The longer the SP, the higher the PPV. The Sen was highest according to RS2 with 4-years SP; however, declined if the SP was 12-years. The coding algorithm with highest PPV and Sen was algorithm “ ≥ 4 outpatient or ≥ 1 inpatient codes” and “ ≥ 2 outpatient or ≥ 1 inpatient codes,” respectively.

**Table 2:** Performance of 6 coding algorithms using *ICD-10-CM* codes for years 2016–2019 to identify people with hepatitis B virus infection according to 3 reference standards (RSs) and 2 study periods

Algorithm	4-years (2016–2019)			12-years (2008–2019)		
	RS1	RS2	RS3	RS1	RS2	RS3
*Positive predictive value*						
1. ≥ 1OP codes	20.5	42.7	85.9	39.0	56.8	86.1
(95% CI)	(16.8–24.2)	(38.2–47.3)	(82.7–89.1)	(34.5–43.5)	(52.3–61.4)	(82.9–89.3)
2. ≥ 2 OP codes	20.2	44.5	87.3	38.9	58.6	87.6
(95% CI)	(16.3–24.1)	(39.7–49.3)	(84.1–90.6)	(34.2–43.6)	(53.9–63.4)	(84.4–90.8)
3. ≥ 3 OP codes	20.7	47.4	89.6	40.1	61.9	89.9
(95% CI)	(16.6–24.9)	(42.3–52.5)	(86.5–92.8)	(35.0–45.1)	(56.9–66.8)	(86.8–93.0)
4. ≥ 2OP or ≥ 1IP codes	20.4	42.3	86.6	38.4	56.2	88.5
(95% CI)	(16.7–24.1)	(37.8–46.8)	(83.4–89.7)	(34.0–42.8)	(51.7–60.7)	(85.6–91.4)
5. ≥ 3OP or ≥ 1IP codes	21.1	44.8	88.6	39.6	59.0	90.8
(95% CI)	(17.2–25.0)	(40.0–49.5)	(85.6–91.7)	(34.9–44.2)	(54.3–63.7)	(88.0–93.5)
6. ≥ 4OP or ≥ 1IP codes	20.9	45.6	89.3	39.9	59.9	91.5
(95% CI)	(17.0–24.9)	(40.8–50.5)	(86.3–92.3)	(35.1–44.7)	(55.1–64.7)	(88.8–94.3)
*Sensitivity*						
1. ≥ 1OP codes	63.7	78.5	72.6	59.6	65.5	53.6
(95% CI)	(55.9–71.5)	(73.4–83.7)	(68.9–76.4)	(54.0–65.2)	(60.8–70.2)	(50.0–57.3)
2. ≥ 2 OP codes	56.8	74.1	66.9	53.9	61.2	49.4
(95% CI)	(48.8–64.9)	(68.6–79.6)	(62.9–70.8)	(48.2–59.5)	(56.4–66.0)	(45.8–53.0)
3. ≥ 3 OP codes	52.1	70.4	61.3	49.5	57.6	45.3
(95% CI)	(44.0–60.2)	(64.8–76.1)	(57.2–65.4)	(43.8–55.2)	(52.7–62.5)	(41.7–48.9)
4. ≥ 2OP or ≥ 1IP codes	64.4	78.9	74.3	59.6	65.7	56.0
(95% CI)	(56.6–72.2)	(73.9–84.0)	(70.6–78.0)	(54.0–65.2)	(61.1–70.4)	(52.4–59.6)
5. ≥ 3OP or ≥ 1IP codes	61.0	76.5	69.6	56.2	63.2	52.5
(95% CI)	(53.1–68.9)	(71.2–81.8)	(65.8–73.5)	(50.6–61.9)	(58.4–68.0)	(48.9–56.2)
6. ≥ 4OP or ≥ 1IP codes	57.5	74.1	66.7	53.9	60.9	50.3
(95% CI)	(49.5–65.6)	(68.6–79.6)	(62.7–70.7)	(48.2–59.5)	(56.1–65.7)	(46.7–54.0)

*IP* inpatients, *OP* outpatient

RS1: having positive results of laboratory test HBsAg or HBeAg

RS2: having RS1 or having prescriptions of anti-HBV drugs

RS3: having RS1 or RS2 or having HBV textual diagnosis recorded in electrical medical records

Table 3 illustrates the PPV and Sen for 6 coding algorithms for identifying people with HCV according to 3 RSs and 2 SPs and Additional file 1: Table S2 presents the numerators and denominators for each PPV and Sen. The PPV was lowest according to RS1 and highest according to RS3. The longer the SP, the higher the PPVs. The Sen was highest according to RS2 compared to those according to RS1 and RS3. The longer the SP, the lower the Sen. The coding algorithm with highest PPV and Sen was “ ≥ 3 outpatient codes” and “ ≥ 2 outpatient or ≥ 1 inpatient codes,” respectively.

**Table 3:** Performance of 6 coding algorithms using *ICD-10-CM* codes for years 2016–2019 to identify people with hepatitis C virus infection according to 3 reference standards (RSs) and 2 study periods

Algorithm	4-years (2016–2019)			12-years (2008–2019)		
	RS1	RS2	RS3	RS1	RS2	RS3
*Positive predictive value*						
1. ≥ 1OP codes	37.3	55.6	94.4	66.7	82.1	94.8
(95% CI)	(32.1–42.6)	(50.1–61.0)	(92.0–96.9)	(61.5–71.8)	(77.9–86.3)	(92.3–97.2)
2. ≥ 2 OP codes	38.9	59.0	96.2	67.6	84.3	96.6
(95% CI)	(33.3–44.5)	(53.4–64.7)	(94.1–98.4)	(62.2–72.9)	(80.1–88.5)	(94.5–98.7)
3. ≥ 3 OP codes	40.2	62.0	97.8	69.7	87.5	97.8
(95% CI)	(34.4–46.1)	(56.2–67.8)	(96.0–99.5)	(64.3–75.2)	(83.5–91.4)	(96.0–99.5)
4. ≥ 2OP or ≥ 1IP codes	37.7	55.2	93.8	65.9	80.7	96.1
(95% CI)	(32.5–42.9)	(49.9–60.5)	(91.2–96.4)	(60.8–70.9)	(76.5–84.9)	(94.1–98.2)
5. ≥ 3OP or ≥ 1IP codes	38.5	57.1	95.0	67.5	83.0	97.2
(95% CI)	(33.1–43.8)	(51.7–62.6)	(92.5–97.4)	(62.4–72.7)	(78.8–87.1)	(95.3–99.0)
6. ≥ 4OP or ≥ 1IP codes	39.0	58.4	95.4	67.9	83.6	97.7
(95% CI)	(33.5–44.5)	(52.8–63.9)	(93.1–97.8)	(62.6–73.1)	(79.5–87.8)	(96.0–99.4)
*Sensitivity*						
1. ≥ 1OP codes	73.3	80.4	75.2	63.3	67.7	58.5
(95% CI)	(66.6–80.1)	(75.2–85.6)	(71.0–79.4)	(58.2–68.5)	(63.1–72.3)	(54.3–62.7)
2. ≥ 2 OP codes	69.1	77.2	69.3	58.1	62.8	53.9
(95% CI)	(62.0–76.1)	(71.7–82.7)	(64.8–73.8)	(52.8–63.3)	(58.1–67.6)	(49.6–58.2)
3. ≥ 3 OP codes	66.1	75.0	65.1	55.4	60.3	50.5
(95% CI)	(58.8–73.3)	(69.3–80.7)	(60.5–69.7)	(50.2–60.7)	(55.5–65.1)	(46.2–54.8)
4. ≥ 2OP or ≥ 1IP codes	77.0	83.0	77.6	65.1	69.2	61.7
(95% CI)	(70.6–83.4)	(78.1–88.0)	(73.6–81.7)	(60.0–70.2)	(64.7–73.8)	(57.6–65.9)
5. ≥ 3OP or ≥ 1IP codes	73.9	80.8	74.0	62.8	66.9	58.7
(95% CI)	(67.2–80.6)	(75.7–86.0)	(69.7–78.2)	(57.6–67.9)	(62.3–71.6)	(54.5–62.9)
6. ≥ 4OP or ≥ 1IP codes	72.1	79.5	71.5	60.7	64.9	56.8
(95% CI)	(65.3–79.0)	(74.2–84.8)	(67.1–75.9)	(55.5–65.9)	(60.2–69.6)	(52.5–61.0)

*IP*  inpatients, *OP*  outpatient

RS1: having positive results of laboratory test Anti-HCV

RS2: having RS1 or having prescriptions of anti-HCV medications

RS3: having RS1 or RS2 or having HCV textual diagnosis recorded in electrical medical records

## Discussion

The findings of this study suggest that the more components used as the RS and the longer the period observed for RS the higher the PPV for identifying people with HBV or HCV. On the contrary, the addition of textual diagnosis as RS and longer period of study for RS would reduce the Sen for identifying people with HBV or HCV. Furthermore, the performance of coding algorithm was better for identifying people with HCV than those with HBV.

**Fig. 1:**
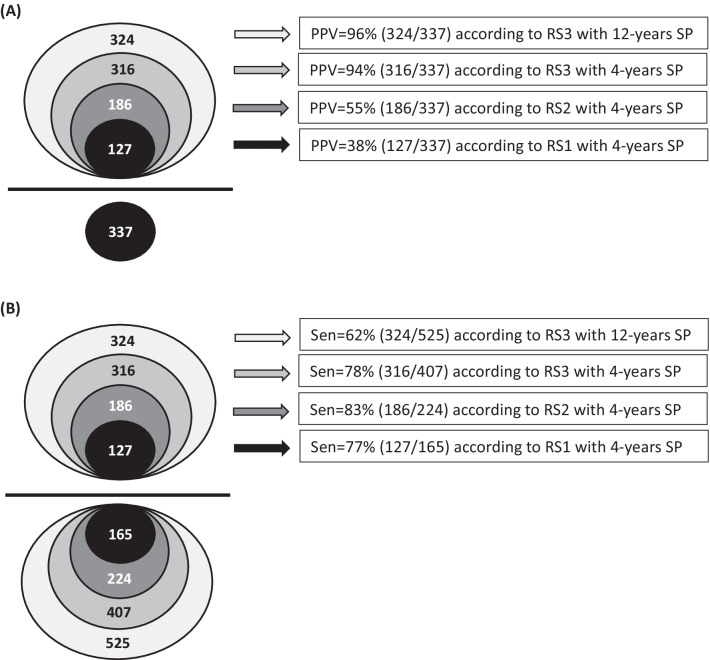
Numerator and denominator for **A** positive predictive values (PPV) and **B** sensitivities (Sen) according to different reference standards (RSs) and study periods (SPs) using coding algorithm “ ≥ 2 outpatients or ≥ 1 inpatients codes” for years 2016–2019 as an example

With regard to the changes in PPV according to different RSs and OPs, as shown in Fig. 1A: the number of participants in denominator for PPV was fixed (participants with positive coding algorithm for years 2016–2019); nevertheless, the number of participants in numerator increased as the component of RS increased ant the period observed increased (more participants with confirmed HCV were identified). The PPV was 38% according to RS1 and increased to 55% and 94% according to RS2 and RS3, respectively. That is to say that if we included pharmacy data in addition to laboratory data as RS we could identify additional 59 participants with confirmed HCV. Furthermore, if we included textual diagnosis recorded in EMR in addition to laboratory and pharmacy data as RS we could identify additional 130 participants with confirmed HCV, a huge increase. Most of 130 participants might have positive Anti-HCV test in other hospitals recently or in his/her ‘usual-care’ hospital several years ago and were recorded as textual diagnosis in EMR.

Regarding the changes in Sen, we noted that both the numerator and denominator changed according to different the RSs and OPs as illustrated in Fig. 1B. The number of participants increased in both numerator and denominator was the same (n = 59) from those according to RS1 to those according to RS2. However, from RS2 to RS3, the increase in number of participants was more prominent in denominator (n = 183) than in numerator (n = 130), which resulted in the decline in Sen calculated. A drastic decline was noted from 4-years SP to 12-years SP, in which the number of participants increased was only 8 in numerator and was 118 in denominator. The most plausible explanation was that many physicians did not give HCV-related *ICD-10-CM* codes in outpatient claims data for these patients had textual diagnosis in EMR and were not noted by physicians.

Of 6 coding algorithms we assessed in this study, we found that the addition of inpatient codes to those used only outpatient codes resulted in a decline in PPV. The decline in PPV was more prominent according to RS2. For example, the PPV was 62% for coding algorithm “ ≥ 3 outpatient codes” for identifying people with HCV and reduced to 55% for coding algorithm “ ≥ 2 outpatient or ≥ 1 inpatient codes” (Table 3). The plausible explanation was that the *ICD-10-CM* codes for discharge diagnosis for inpatient hospitalization were assigned by professional nosologists who had plenty time to review the medical records to find out the textual diagnosis recorded in past history and gave HBV- or HCV-related *ICD-10-CM* codes. These participants with positive coding algorithm could not be confirmed according results of laboratory tests and pharmacy data. The decline in PPV was less prominent according to RS3, which from 98 to 94%, respectively, because most of the participants with positive coding algorithm increased could be confirmed if we include textual diagnosis as RS.

On the contrary, we found that the addition of inpatient codes in coding algorithm to those used only outpatient codes resulted in an increase in Sen. Because the *ICD-10-CM* codes for outpatient diagnosis were given by physicians in the clinics in which physicians might not have enough time to check the results of laboratory tests and pharmacy data or textual diagnosis in past history and therefore did not give HBV- or HCV- related *ICD-10-CM* codes resulted in false negative misclassification. The addition of inpatient diagnosis codes given by nosologists, most of these added participants with positive coding algorithm were true positive and resulted in an increase of Sen.

Compared with the PPV and Sen in previous US studies, the Centers for Disease Control and Prevention Chronic Hepatitis Cohort Study in the United States used an algorithm of two ICD-9 codes separated by ≥ 6 months (including both outpatient and inpatient claims data) had a PPV of 90% for HBV and 92% for HCV and Sen of 58% for HBV and 70% for HCV [13, 15]. The compatible coding algorithm “ ≥ 2 outpatient codes” in this study had a PPV of 87% for HBV and 96% for HCV and Sen of 67% for HBV and 69% for HCV according to RS3 with 4-years observation period. Given the same coding algorithm, the validity of using *ICD-10-CM* codes in Taiwan NHI claims data was similar to the healthcare system claims data in the United States.

Our algorithms exhibited better performance in identifying people with HCV than people with HBV. This was likely because the NHI has covered direct-acting antiviral agents (DAAs) for people with HCV since January 24, 2017 [19]. The physicians were required to provide *ICD-10-CM* codes for people with HCV for prescribing DAAs; thus improve the quality of coding.

The performance of our algorithms were better than previous Taiwan study (using coding algorithm “ ≥ 1 outpatient codes,” in which the PPV was 45% for HBV and 81% for HCV and the Sen was 46% for HBV and 47% for HCV) because of two possible reasons [16]. First, in addition to the results of laboratory tests used by previous study for RS, we added drug prescription and textual diagnosis recorded in EMR as RS and a longer observation period (2016–2019) than in the previous study (laboratory results for one quarter in 2018 as RS). Some of the people with HBV- or HCV-related *ICD-10-CM* codes judged as false positive in previous studies might have been judged as true positive in this study because of more evidence to confirm patients had HBV or HCV. Second, this study was confined to one health care system with three hospitals with relatively high quality of coding, and the previous study covered thousands of hospitals and clinics in Taiwan.

One of the strengths of this study is large sample size. Unlike some previous studies using *ICD* codes to recruit patients, which allowed only PPV estimation [12, 13], in this study, by using proportional random sampling of 10,000 patients, we could calculate not only PPV but also Sen. Second, compared the PPV and Sen according to different RSs and OPs which could provide useful information for future researches in determining the RS and the OP. Third, this study is the first to examine the performance of various coding algorithms using *ICD-10-CM* codes to identify people with HBV and HCV.

Nevertheless, our study also had several limitations. First, this study was confined to a health care system in southern Taiwan, which might affect the generalization to other populations. However, the main findings (better performance for identifying people with HCV than those with HBV) were affected by contextual factor (the reimbursement of DAA). Therefore, we believe that these conclusions may be applicable to other clinical settings in Taiwan. Second, some of the patients might have positive results of laboratory tests or receiving anti-HBV or anti-HCV medication in other hospitals and were not tested and treated in this health care system, rendering them been determined as false negatives in this study. Third, it seemed unfair to use 12-years SP for RS to determine the performance of coding algorithms for 4-years; since most physicians tend to code for the clinical condition that prompted care that day. However, for HBV and HCV, we have higher expectation upon physicians to enquiry patients on the awareness of having HBV or HCV to achieve the goal of eliminating viral hepatitis as a major public health threat by 2030 set by the World Health Organization [20]. Fourth, the use of textual diagnosis of HBV or HCV from past history as one of components for RS might be incorrect due to patient’s recall bias. Fifth, we did not add time interval between outpatient visits in defining 6 algorithms. The coding algorithms used by the US CHeCS requested that the two ICD diagnosis codes should be at least 6 months apart [13, 15].

In conclusion, as the status of HBV and HCV is lifelong, the use of RS should include not only positive results of laboratory tests and pharmacy data, but also textual diagnosis recorded in medical records and the OP should be as long as possible. The findings of this study suggest that the best coding algorithm for identifying people with HBV or HCV was “ ≥ 3 outpatient codes” for PPV and “ ≥ 2 outpatient or ≥ 1 inpatient codes” for Sen. To achieve the best yield of both PPV and Sen, we recommended the optimal coding algorithm was ≥ 2 outpatient or ≥ 1 inpatient codes.

## Supplementary Information

**Additional file 1: Table S1.** Numerator and denominator for calculating positive predictive value and sensitivity in various coding algorithms to identify people with hepatitis B virus infection based on 3 reference standards (RS) and two study periods. **Table S2. **Numerator and denominator for calculating positive predictive value and sensitivity in various coding algorithms to identify people with hepatitis C virus infection based on 3 reference standards (RS) and two study periods.

## Data Availability

The data are available from corresponding author upon reasonable request.

## Abbreviations

DAA: Direct oral antiviral agents
HBV: Hepatitis B virus
HCV: Hepatitis C virus
*ICD*: *International Classification of Diseases*
*ICD-10-CM*: *International Classification of Diseases Tenth Revision Clinical Modification*
NHI: National Health Insurance
OP: Observation period
PPV: Positive predictive value
RECORD: Reporting of studies conducted using observational routinely-collected health data
RS: Reference standard
Sen: Sensitivity
