# Supplementary material for: Validation of coding algorithms for identifying people with viral hepatitis using claims data according to different standard references
Source: BMC Infect Dis. 2022 Mar 4;22:222. doi: 10.1186/s12879-022-07212-w (PMC8897839; doi:10.1186/s12879-022-07212-w)
Supplement: Supplementary file 1 — Additional file 1: Table S1. Numerator and denominator for calculating positive predictive value and sensitivity in various coding algorithms to identify people with hepatitis B virus infection based on 3 reference standards (RS) and two study periods. Table S2. Numerator and denominator for calculating positive predictive value and sensitivity in various coding algorithms to identify people with hepatitis C virus infection based on 3 reference standards (RS) and two study periods. [file 12879_2022_7212_MOESM1_ESM.docx]

**Table S1.** Numerator and denominator for calculating positive predictive value and sensitivity in various coding algorithms to identify people with hepatitis B virus infection based on 3 reference standards (RS) and two study periods

|  |  | 4-years (2016-2019) | | |  | 12-years (2008-2019) | | |
| --- | --- | --- | --- | --- | --- | --- | --- | --- |
| Algorithm |  | RS1 | RS2 | RS3 |  | RS1 | RS2 | RS3 |
|  |  | Positive predictive value | | | | | | |
| 1.  ≥1OP codes | TP | 93 | 194 | 390 |  | 177 | 258 | 391 |
|  | TP+FP | 454 | 454 | 454 |  | 454 | 454 | 454 |
| 2.   ≥2 OP codes | TP | 83 | 183 | 359 |  | 160 | 241 | 360 |
|  | TP+FP | 411 | 411 | 411 |  | 411 | 411 | 411 |
| 3.    ≥3 OP codes | TP | 76 | 174 | 329 |  | 147 | 227 | 330 |
|  | TP+FP | 367 | 367 | 367 |  | 367 | 367 | 367 |
| 4.    ≥2OP or ≥1IP codes | TP | 94 | 266 | 399 |  | 177 | 259 | 408 |
|  | TP+FP | 461 | 461 | 461 |  | 461 | 461 | 461 |
| 5.    ≥3OP or ≥1IP codes | TP | 89 | 233 | 374 |  | 167 | 249 | 383 |
|  | TP+FP | 422 | 422 | 422 |  | 422 | 422 | 422 |
| 6.    ≥4OP or ≥1IP codes | TP | 84 | 218 | 358 |  | 160 | 240 | 367 |
|  | TP+FP | 401 | 401 | 401 |  | 401 | 401 | 401 |
|  |  | Sensitivity | | | | | | |
| 1.  ≥1OP codes | TP | 93 | 194 | 390 |  | 177 | 258 | 391 |
|  | TP+FN | 146 | 247 | 537 |  | 297 | 394 | 729 |
| 2.   ≥2 OP codes | TP | 83 | 183 | 359 |  | 160 | 241 | 360 |
|  | TP+FN | 146 | 247 | 537 |  | 297 | 394 | 729 |
| 3.    ≥3 OP codes | TP | 76 | 174 | 329 |  | 147 | 227 | 330 |
|  | TP+FN | 146 | 247 | 537 |  | 297 | 394 | 729 |
| 4.    ≥2OP or ≥1IP codes | TP | 94 | 266 | 399 |  | 177 | 259 | 408 |
|  | TP+FN | 146 | 247 | 537 |  | 297 | 394 | 729 |
| 5.    ≥3OP or ≥1IP codes | TP | 89 | 233 | 374 |  | 167 | 249 | 383 |
|  | TP+FN | 146 | 247 | 537 |  | 297 | 394 | 729 |
| 6.    ≥4OP or ≥1IP codes | TP | 84 | 218 | 358 |  | 160 | 240 | 367 |
|  | TP+FN | 146 | 247 | 537 |  | 297 | 394 | 729 |
| RS1：positive results of laboratory test  RS2：positive results of laboratory test or having prescriptions of anti-HBV drugs  RS3：positive results of laboratory test or having prescriptions of anti-HBV drugs or having HBV textual diagnosis  TP: true positive; FP: false positive; FN: false negative; TN: true negative; IP: inpatient; OP: outpatient | | | | | | | | |

**Table S2.** Numerator and denominator for calculating positive predictive value and sensitivity in various coding algorithms to identify people with hepatitis C virus infection based on 3 reference standards (RS) and two study periods

|  |  | 4-years (2016-2019) | | |  | 12-years (2008-2019) | | |
| --- | --- | --- | --- | --- | --- | --- | --- | --- |
| Algorithm |  | RS1 | RS2 | RS3 |  | RS1 | RS2 | RS3 |
|  |  | Positive predictive value | | | | | | |
| 1.  ≥1OP codes | TP | 121 | 180 | 306 |  | 216 | 266 | 307 |
|  | TP+FP | 324 | 324 | 324 |  | 324 | 324 | 324 |
| 2.   ≥2 OP codes | TP | 114 | 173 | 282 |  | 198 | 247 | 283 |
|  | TP+FP | 293 | 293 | 293 |  | 293 | 293 | 293 |
| 3.    ≥3 OP codes | TP | 109 | 168 | 265 |  | 189 | 237 | 265 |
|  | TP+FP | 271 | 271 | 271 |  | 271 | 271 | 271 |
| 4.    ≥2OP or ≥1IP codes | TP | 127 | 186 | 316 |  | 222 | 272 | 324 |
|  | TP+FP | 337 | 337 | 337 |  | 337 | 337 | 337 |
| 5.    ≥3OP or ≥1IP codes | TP | 122 | 181 | 301 |  | 214 | 263 | 308 |
|  | TP+FP | 317 | 317 | 317 |  | 317 | 317 | 317 |
| 6.    ≥4OP or ≥1IP codes | TP | 119 | 178 | 291 |  | 207 | 255 | 298 |
|  | TP+FP | 305 | 305 | 305 |  | 305 | 305 | 305 |
|  |  | Sensitivity | | | | | | |
| 1.  ≥1OP codes | TP | 121 | 180 | 306 |  | 216 | 266 | 307 |
|  | TP+FN | 165 | 224 | 407 |  | 341 | 393 | 525 |
| 2.   ≥2 OP codes | TP | 114 | 173 | 282 |  | 198 | 247 | 283 |
|  | TP+FN | 165 | 224 | 407 |  | 341 | 393 | 525 |
| 3.    ≥3 OP codes | TP | 109 | 168 | 265 |  | 189 | 237 | 265 |
|  | TP+FN | 165 | 224 | 407 |  | 341 | 393 | 525 |
| 4.    ≥2OP or ≥1IP codes | TP | 127 | 186 | 316 |  | 222 | 272 | 324 |
|  | TP+FN | 165 | 224 | 407 |  | 341 | 393 | 525 |
| 5.    ≥3OP or ≥1IP codes | TP | 122 | 181 | 301 |  | 214 | 263 | 308 |
|  | TP+FN | 165 | 224 | 407 |  | 341 | 393 | 525 |
| 6.    ≥4OP or ≥1IP codes | TP | 119 | 178 | 291 |  | 207 | 255 | 298 |
|  | TP+FN | 165 | 224 | 407 |  | 341 | 393 | 525 |
| RS1：positive results of laboratory test  RS2：positive results of laboratory test or having prescriptions of anti-HBV drugs  RS3：positive results of laboratory test or having prescriptions of anti-HBV drugs or having HBV textual diagnosis  TP: true positive; FP: false positive; FN: false negative; TN: true negative; IP: inpatient; OP: outpatient | | | | | | | | |
